# Mapping Excited-State Decay Mechanisms in Acetylacetone by Sub-20 fs Time-Resolved Photoelectron Spectroscopy

**DOI:** 10.1021/jacs.5c06327

**Published:** 2025-08-13

**Authors:** Stefano Severino, Flavia Aleotti, Lorenzo Mai, Aurora Crego, Fabio Medeghini, Fabio Frassetto, Luca Poletto, Matteo Lucchini, Francesco Segatta, Maurizio Reduzzi, Mauro Nisoli, Artur Nenov, Rocío Borrego-Varillas

**Affiliations:** † Dipartimento di Fisica, 18981 Politecnico di Milano, Piazza Leonardo da Vinci 32, Milano 20133, Italy; ‡ Department of Industrial Chemistry “Toso Montanari”, 9296 University of Bologna, Via Piero Gobetti 86, Bologna 40129, Italy; § Institute of Photonics and Nanotechnologies, CNR (CNR-IFN), Piazza Leonardo da Vinci 32, Milano 20133, Italy; ∥ Grupo de Investigación en Aplicaciones del Láser y Fotónica, Departamento de Física Aplicada, 16779 Universidad de Salamanca, Salamanca E-37008, Spain; ⊥ Institute for Photonics and Nanotechnologies, IFN-CNR, via Trasea 7, Padova 35131, Italy

## Abstract

Excited-State Intramolecular Hydrogen Transfer (ESIHT) is one of the fastest chemical reactions, occurring on the order of tens of femtoseconds and playing a critical role in light-driven biological processes and technological applications. Here, we investigate the early stages of coupled nuclear-electron dynamics using acetylacetone (AcAc) as a model system exhibiting ESIHT. We employ ultraviolet-extreme ultraviolet (UV-XUV) time-resolved photoelectron spectroscopy (tr-PES) with sub-20 fs resolution in combination with high-level dynamically correlated simulations (CASPT2) to map the electronic relaxation pathways and vibrational modes driving this process. Our results provide distinct spectroscopic signatures of ESIHT occurring within the first 20 fs and resolve the active vibrational modes, showing the intricate evolution of the electronic and nuclear degrees of freedom. Moreover, the analysis reveals the key role of ultrafast intersystem crossing (ISC) to triplet states in modulating the excited-state dynamics and its implications for the overall relaxation pathways. These findings refine our understanding of the photochemistry of AcAc and suggest general principles that can be applied to similar conjugated systems.

## Graphical abstract

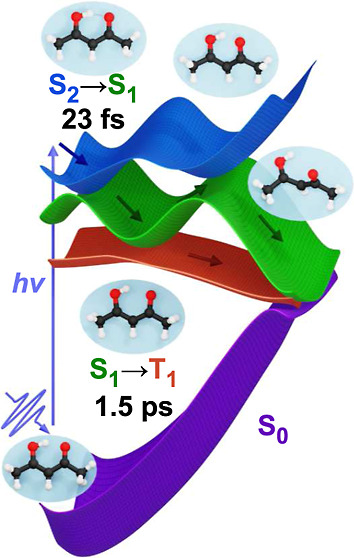

## 1 Introduction

The process of Excited-State Intramolecular Hydrogen Transfer (ESIHT), an example of a prototropic tautomerization, stands out as one of the fastest excited-state phenomena, occurring within a remarkably short time frame of a few tens of femtoseconds.^1–6^ This isomerization process represents the first step in a broad spectrum of light-triggered biological functions^7–9^ and technological endeavors. Acetylacetone (AcAc) serves as an archetypal system presenting ESIHT dynamics and keto–enol tautomerism.^10,11^ In gas-phase AcAc, the keto–enol tautomerism is dominated by the enolic form,^10^ in which the intramolecular hydrogen bond gives rise to a cyclic structure. The presence of a central six-membered ring constitutes a crucial structural characteristic shared by numerous molecules. The hydrogen atom sealing the central ring in these molecules is strategically positioned for transfer between the two oxygen atoms.

The ultrafast dynamics of AcAc has been the subject of intense research in the literature,^12–19^ yet a complete understanding remains elusive. One of the earliest time-resolved investigations was conducted by Zewail and co-workers using ultrafast electron diffraction,^12^ concluding that the dominant reaction pathway involves elimination of an OH radical via the T_1_(ππ*) triplet state, with a characteristic decay time of about 247 ps. The proposed mechanism involved rapid internal conversion (IC) from the initially excited S_2_(ππ*) state to the lower-lying S_1_(nπ*) state, followed by intersystem crossing (ISC) to T_1_(ππ*), although these intermediate steps could not be directly observed. Poisson et al. further explored the system using time-resolved photoelectron imaging.^13^ A short time constant of about 70 fs was associated with the S_2_(ππ*) state departing the Franck–Condon region, while a second time constant of 1.4 ps was attributed to IC to the S_1_(nπ*) state. A considerably slower process, lasting up to 80 ps, was subsequently attributed to ISC to lower-lying triplet states. Bhattacherjee et al. employed ultrafast soft X-ray transient absorption spectroscopy, challenging previous ISC time scales by measuring ISC from S_1_(nπ*) to T_1_(ππ*) occurring much faster, around 1.5 ps.^16^ Although they could not directly observe the IC from S_2_ to S_1_ due to spectral overlap, it was inferred to occur on a sub-100 fs time scale. This faster picture of ISC was also supported by Squibb et al.^17^ Kotsina et al. used time-resolved photoelectron imaging with a VUV probe, concluding that the S_2_ to S_1_ passage occurs with a time constant below 50 fs (limited by the time resolution of the experiment), followed by ISC from S_1_ to T_1_ in 1.6 ps and relaxation to the minimum of T_1_ in 20 ps.^18^

In summary, while the S_2_/S_1_ crossing has been inferred to occur within 50 fs, direct spectroscopic evidence of this ultrafast process is missing due to the lack of sufficient temporal resolution in previous studies.^13,14,16–19^ After ultrafast transfer to S_1_, the system can either undergo direct S_1_ → S_0_ IC or ISC to the triplet manifold, which then survives for hundreds of picoseconds before decaying back to the ground state.^17,18^ Although recent works identify the ISC as the major relaxation channel, the time scale in which the triplet manifold is populated is still debated. While early time-resolved experiments on AcAc proposed ISC to occur in tens to a few hundreds of picoseconds,^12,13^ more recent studies suggest it to be much faster (∼1.5 ps), attributing the long time constant to either T_1_ relaxation or T_1_ → S_0_ decay.^16–18^

By means of ultraviolet-extreme ultraviolet (UV-XUV) transient photoelectron spectroscopy (tr-PES) with sub-20 fs time resolution,^20^ exceeding by 1 order of magnitude that of previous studies on AcAc, we capture the evolution of the coupled electron–nuclear dynamics and the associated vibrational modes from the bright S_2_ state to the ground state. Specifically, we determine the time scale of the S_2_/S_1_ crossing along the ESIHT coordinate and identify a spectral signature of the triplet state. In addition, we detect high-frequency vibrational modes coherently activated on S_2_ and S_1_, which favorably tune the singlet–triplet gap to boost the ISC rate. High-level theoretical simulations (CASPT2) of the photoinduced dynamics and of the resulting tr-PES signals show excellent agreement with the experimental data, allowing us to dig deeper into AcAc deactivation dynamics and refine our understanding of how the electronic and nuclear degrees of freedom evolve during ESIHT. These insights expand our knowledge of ultrafast photochemical processes in conjugated systems and suggest potential applications for controlling similar reactions in biorelevant molecules.

## 2 Results and Discussion

### 2.1 Time-Resolved Photoelectron Spectra

The sketch of the experiment is shown in Figure 1(a): a UV pulse, with a central photon energy of 4.7 eV, prepares an electronic wave packet in the S_2_ state, while a single XUV pulse, centered at 24.7 eV, probes its evolution by monitoring the kinetic energy of the ejected photoelectrons as a function of the pump–probe delay (further details on the experimental setup are given in Section 4.1 and in the Supporting Information). The same time-resolved experiment was simulated by means of CASPT2 calculations of ionization energies on top of ab initio surface-hopping molecular dynamics started on the bright state (details in Section 4.2). In panels (b) and (c), we present the resulting experimental and simulated tr-PES traces, respectively. The experimental data reveal an immediate emergence of a low binding energy signal (3.5 eV–5 eV) that decays over tens of femtoseconds, while, simultaneously, a new blue-shifted signal appears (5 eV–6.5 eV). This evolution is captured by the center of mass (CM) calculated between 3 and 8 eV (white curves in Figure 1(b) and (c)), which shows a quick relaxation of the system in the first 50 fs. At delays larger than 250 fs, the signal centered at 5.75 eV starts decaying while shifting toward larger binding energies. The sub-20 fs time resolution of our experimental setup reveals impulsively excited coherent oscillations modulating this signal, which can be exploited to gain insights into the excited-state vibrational dynamics of the system. A global analysis, in which the rise and decay time constants were left free (see details in Section S2 of the Supporting Information), was performed on both the experimental and theoretical maps. The resulting associated spectra are shown in Figure 2(a) and (b). A first spectrum (labeled as A_1_ in Figure 2(a)), with an associated time constant of τ_1_ = 23 ± 2 fs, is centered at 4.2 eV. The second spectral signature (A_2_), centered at 5.6 eV, decays with a time constant τ_2_ = 1520 ± 135 fs. Finally, a long-living signal (A_3_), decaying beyond the time window of the data, emerges at larger binding energies with a rise time of 1610 ± 125 fs, which is statistically indistinguishable from τ_2_. This time constant agrees well with the value attributed to ISC in recent works.^16–18^ We note that these measurements were optimized to capture the early time dynamics, and therefore, the temporal window was limited to 700 fs, which led to a larger uncertainty in the determination of τ_2_ and τ_3_. A long-time scale measurement is reported in Figure S2 of the Supporting Information, confirming the extracted time constants. The analysis of the simulated trace yields similar results, with only a discrepancy in the A_2_ decay time constant: the simulated spectroscopic signature of S_1_ decays faster than in the experiment (903 fs vs 1520 fs, see Section S2 of Supporting Information), an aspect that will be addressed in more detail in Section 2.5.

**Figure 1:**
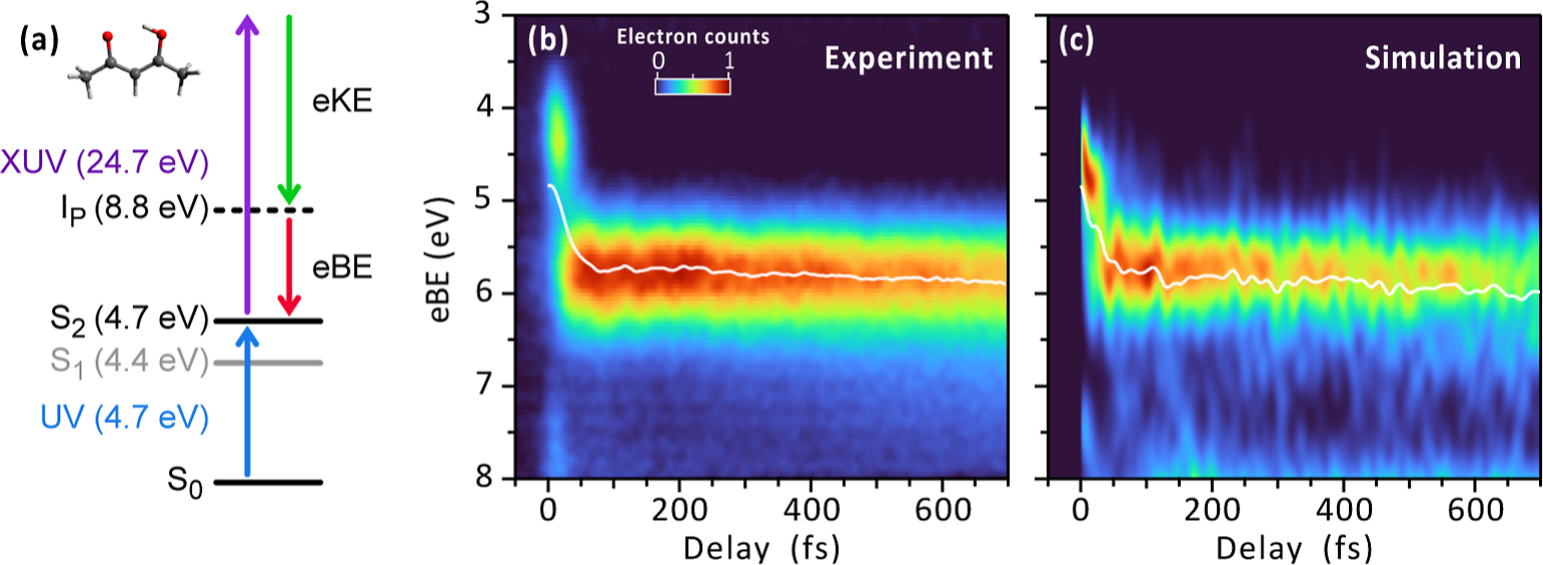
Tr-PES of acetylacetone. (a) Diagram of the experiment: a UV pump pulse excites the system in the S_2_ level and an XUV probe ionizes it; the resulting electron Binding Energy (eBE), or equivalently the electron Kinetic Energy (eKE), tracks the induced dynamics. Experimental (b) and simulated (c) evolution of the eBE as a function of the pump and probe delay. Both traces show a rapid increase of the eBE in the first tens of femtoseconds, followed by a higher binding energy signal exhibiting a periodic chemical shift. In white, superimposed to the traces, is the evolution of the center of mass.

**Figure 2:**
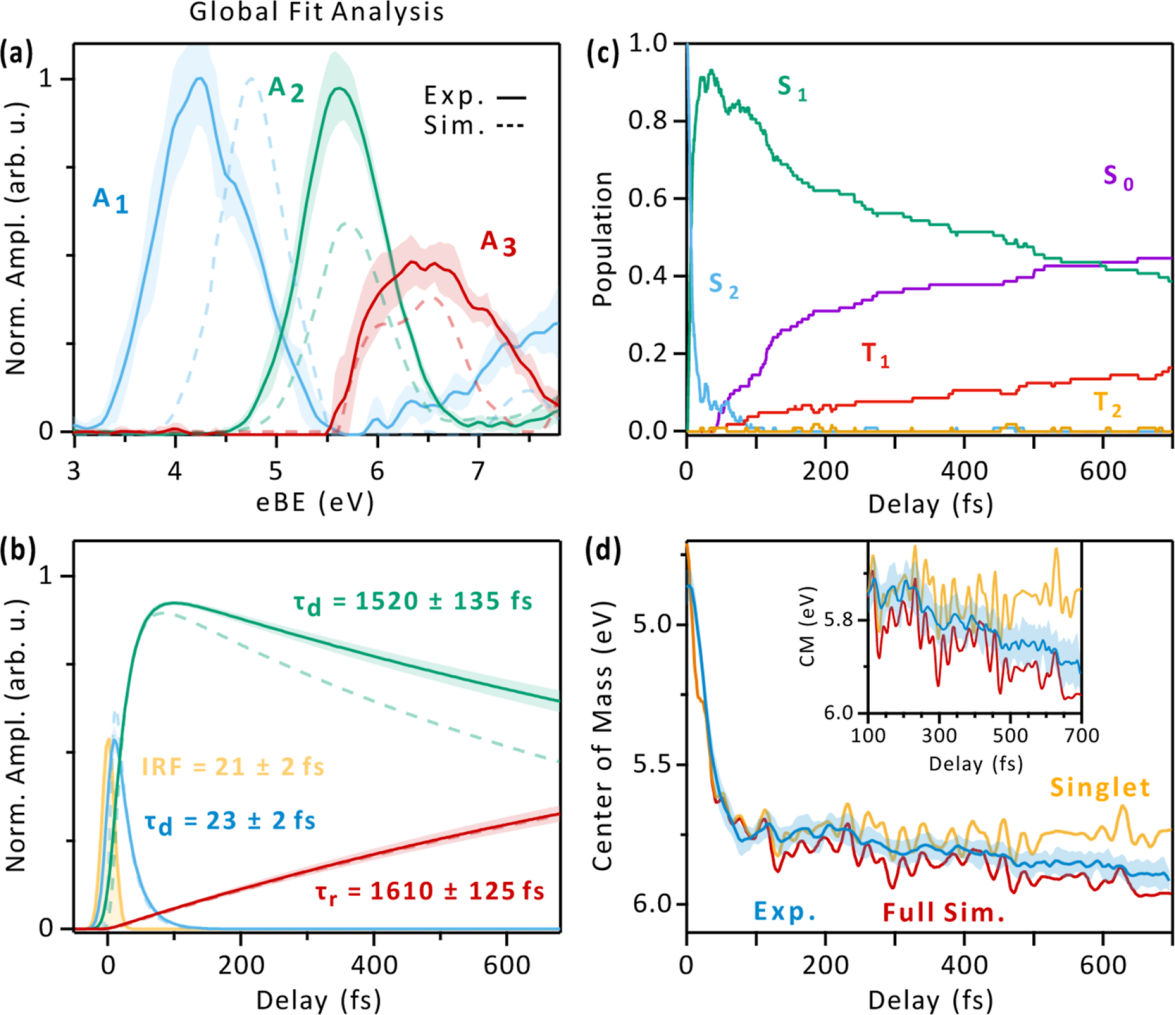
Analysis of the ultrafast dynamics. (a) Spectral and (b) temporal amplitudes of the three components resulting from the global fit analysis, on the experimental (continuous lines) and simulated traces (dotted lines). The relevant fitting parameters of the experimental trace are displayed (further details in Section S2 of the Supporting Information). The IRF of the experiment resulting from the global fit, which matches the results of the independent two-color Ar photoionization measurement (Section S1 of Supporting Information), is marked in yellow. (c) Population dynamics from the simulation; (d) evolution of the center of mass (CM) in the experimental (blue) and simulated maps and its zoom in the inset (red: full simulation; yellow: simulation with singlet states only). In (a), (b), and (d), the shaded area represents the standard deviation of the experimental results.

### 2.2 Internal Conversion Involving ESIHT Proceeds in 23 fs

By accurately replicating the experimental fingerprints, we were able to correlate the spectral features with their respective states. The vertical arrows in Figure 4(b) show the expected binding energies of the PES signals at relevant geometries. At the Franck–Condon (FC) region, the bright S_2_ state (ππ*) is expected to give a PES contribution at 4.16 eV. We thus assign the A_1_ signal to the evolution of S_2_. Figure 2(c) shows the population dynamics calculated by the theory for the singlet and triplet states. We observe that S_2_ is depopulated quickly after excitation, with most of the electronic population reaching the first excited state (S_1_) within a few tens of fs. The time constant τ_1_ = 23 ± 2 fs is therefore assigned to this conical intersection (CI) passage. As will be explained in detail in Section 2.5, the S_2_/S_1_ crossing occurs in the vicinity of the FC point (see electronic-state energies in Table S3 and geometrical parameters in Figure S5), and it is reached from the S_0_ equilibrium geometry mainly along H transfer and C–C/C–O bond length alternation (BLA) coordinates. This suggests the involvement of an ESIHT process, which is experimentally confirmed by information gained on the nuclear deformations driving the S_2_/S_1_ CI through the coherent oscillation analysis (Section 2.4).

### 2.3 Spectroscopic Signature of a Triplet-Mediated Decay Channel

Focusing on the A_3_ signal, its temporal evolution matches the increase in population in the T_1_ state (red curve in Figure 2(c)), and its spectral position agrees with the PES signal expected from the T_1_ state at its minimum (Figure 4(b)). To convincingly assign the origin of A_3_, we repeated the nonadiabatic dynamics and spectroscopy simulation, excluding the triplet states. In Figure 2(d), we show the behavior of the CM of the experimental trace (blue curve) and of the simulated traces with (red) and without (yellow) triplets. Starting from 250 fs, both the experimental trace and the trace obtained considering the triplets shift toward larger binding energies. This is a consequence of the appearance of a new signal centered at 6.5 eV (A_3_). In contrast, the simulation involving only the singlet states maintains a constant average binding energy, therefore diverging from the others as time progresses (see the zoomed-in inset of Figure 2(d)). This comparison demonstrates that the appearance of A_3_ is related to the flow of population from the S_1_ state to the triplet manifold through ISC, in agreement with the hypothesis of a fast transfer of population to the triplet manifold proposed by most recent studies on AcAc.^16–18^ To further support this observation, the disentanglement of the contributions of singlet and triplet states to the simulated tr-PES signal (Figure S10) clearly shows that the A_3_ component is ascribable exclusively to T_1_. Importantly, we note that the T_1_ contribution initially overlaps with the S_1_ signal around 5.75 eV, but over time, it shifts toward 6.5 eV (see also Figure S2 in the Supporting Information). This is in agreement with an ISC event taking place around S_1_ minimum (where T_2_ and T_1_ ionization peaks are mostly overlapping with the S_1_ signal, see Table S4), followed by a subsequent relaxation toward the T_1_ minimum. The global fit analysis on the experimental trace assigns an exponential rise to the A_3_ of 1610 ± 125 fs, in good agreement with the results from the simulation, therefore unambiguously identifying the timings of the ultrafast ISC in AcAc relaxation dynamics.

### 2.4 Coherent Vibrational Dynamics Driving ESIHT and ISC

Both the experimental and simulated traces exhibit an oscillatory modulation, clearly visible in the evolution of the center of mass (CM) of the photoelectron signal (Figure 2(d) and its background-subtracted counterpart in Figure 3(a)). This oscillatory behavior provides valuable insights into the vibrational modes that govern the relaxation pathways of the system. Figure 3(b) shows the experimental and simulated Fourier transform (FT) analysis.

**Figure 3:**
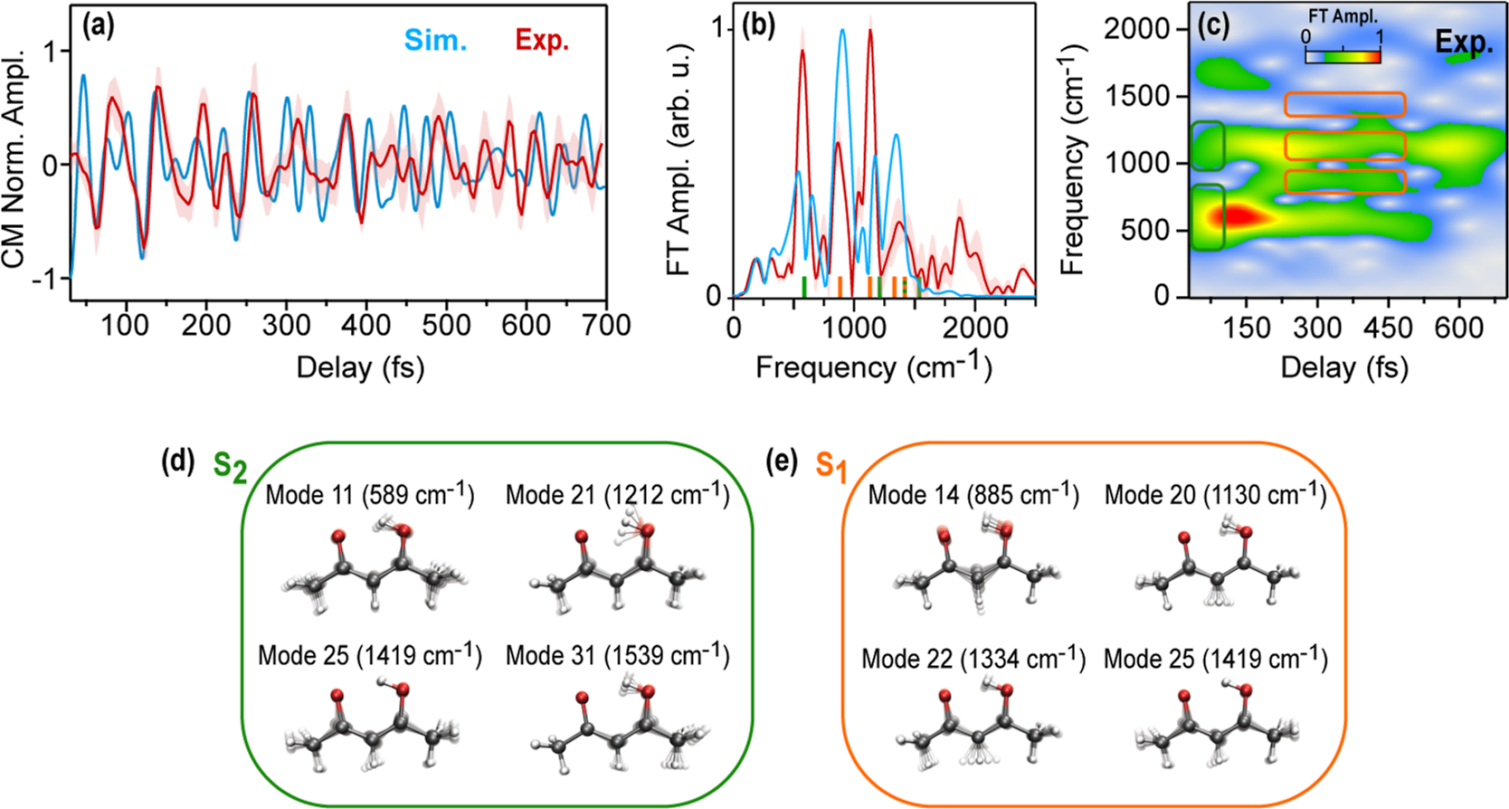
Vibrational coherences. Experimental (red) and simulated (blue) background-subtracted CM calculated between 5.3 and 6.5 eV (a) and its static Fourier Transform (FT) (b). In (c), the Gabor analysis of the experimental data was performed with a 300 fs-wide moving window. The vibrational modes associated with the S_2_ and S_1_ surfaces are shown in (d) and (e) and are highlighted in panel (c) in green and orange, respectively.

To interpret the frequency peaks from the Fourier analysis in terms of excited-state vibrational modes, we performed an S_1_ geometry optimization and frequency calculation (XMS-CASPT2/SA5-CASSCF­(10,8)/cc-pVDZ). The S_1_ and S_2_ gradients at the Franck–Condon point were projected onto the normal modes to identify those most strongly activated in each state. This allows us to attribute the peaks in the FT to vibrational modes conserving planar symmetry, which are activated either on the S_2_ or the S_1_ surfaces (highlighted, respectively, in green and orange in Figure 3, see Section S4.2 of the Supporting Information for further details). A Gabor transform (Figure 3(c)) provides a clear and comprehensive depiction of the temporal evolution of the vibrational modes and, by extension, of the nuclear dynamics.

Immediately after pump excitation, the strongest contributions are from mode 11 (589 cm^–1^—oscillation period: 57 fs) and mode 21 (1212 cm^–1^–28 fs). These modes, associated with an oscillatory motion of the central hydrogen atom (ESIHT coordinate) and C–C/C–O BLA typical of excited polyconjugated molecules, tune the S_2_–S_1_ energy gap and drive the system to S_2_/S_1_ minimum energy conical intersection (MECI) on a few-fs time scale. We note that mode 11, activated in the S_2_ state, has a lifetime considerably longer than the state itself. This is due to a memory effect typical of low-frequency bending modes, whose vibrations can survive IC. In the case of AcAc, this mode is strongly activated upon excitation to S_2_ (as shown by gradient projections) but not on S_1_, indicating that vibrational energy acquired on S_2_ carries over to S_1_ and remains coherent for several hundred femtoseconds, further sustained by the lack of solvent damping.

As the pump–probe delay increases, mode 11 decays, while a new higher-frequency contribution appears at 885 cm^–1^ (mode 14–38 fs). Simultaneously, we can distinguish frequencies matching those of mode 20 (1130 cm^–1^–30 fs) and mode 25 (1419 cm^–1^–23 fs), respectively. All these modes are associated with angle deformations and ring opening of the conjugated unit, which reflect the relaxation toward the S_1_ minimum (see Figures 4 and S5). At delays larger than 500 fs, the strongest contributions can be assigned to modes 14 and 20.

**Figure 4:**
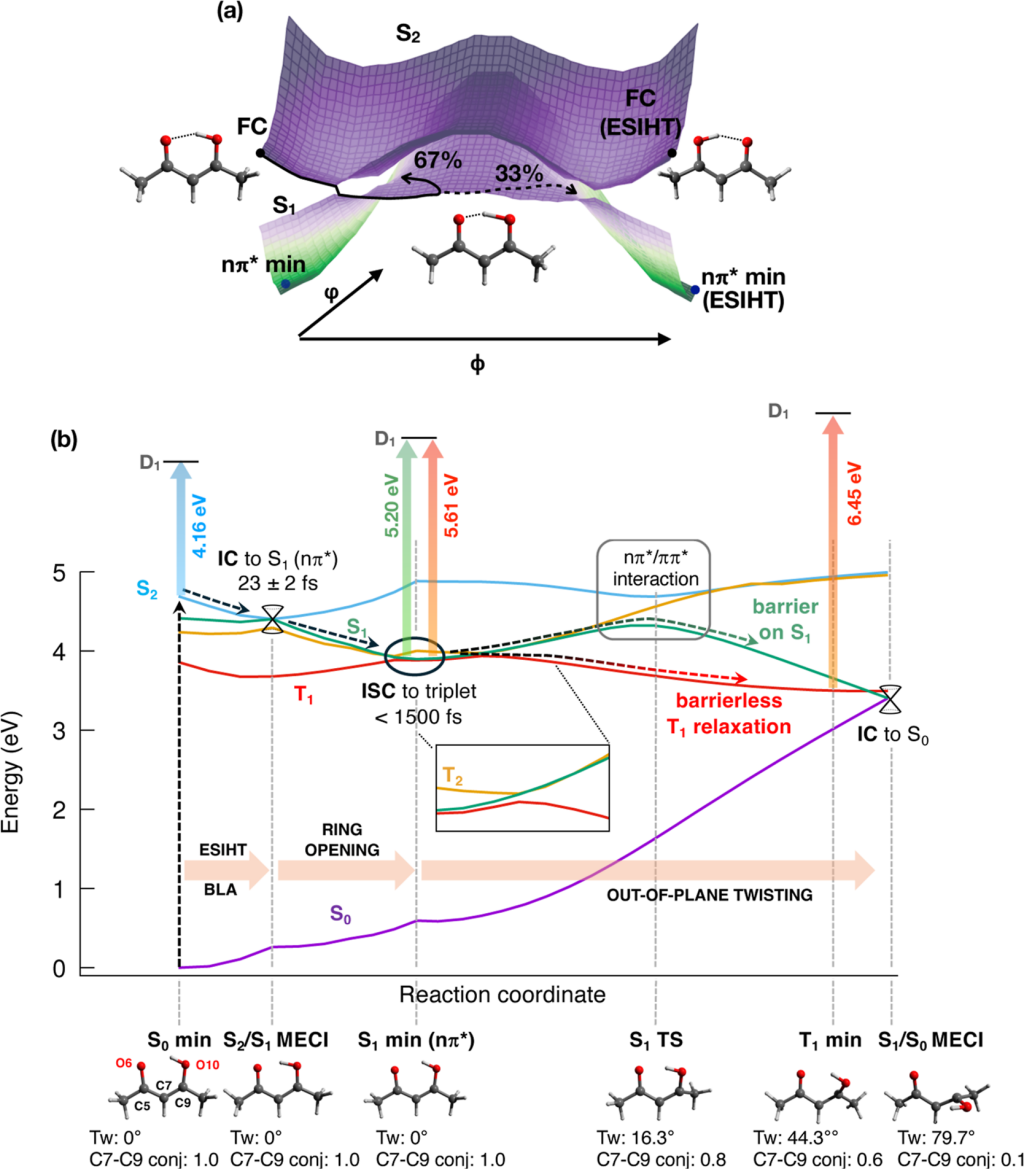
Acetylacetone deactivation mechanism. (a) Potential energy surfaces of S_1_ and S_2_ in the space of ESIHT + BLA coordinates (ϕ) and ring opening (φ): after S_2_ → S_1_ decay, ESIHT is interrupted in most of the cases (67%), and trajectories deviate along φ to reach the S_1_ minimum; (b) potential energy profiles for AcAc along geodesic interpolations between key geometries (S_0_ min → S_2_/S_1_ MECI → S_1_ min → S_1_/S_0_ MECI, via S_1_ TS and T_1_ min). The inset magnifies the vicinity of S_1_ min. The predicted PES signals (binding energies, in eV) from critical points are shown as vertical arrows. The most activated vibrations in each step of the deactivation path are shown as horizontal arrows. Geometrical parameters: Tw = O10–C9–C5–O6 dihedral; C7–C9 conjugation = cosine of the angle between the p orbitals of C7 and C9 (1 = complete conjugation, 0 = orthogonal orbitals, no conjugation).

Further investigation of the four S_1_ active modes revealed that each of them is associated with a reorganization energy of ca. 0.1 eV when moving from S_2_/S_1_ MECI to the S_1_ minimum, and that the same modes tune the singlet–triplet gap (Figure S8), leading to a crossing in the immediate proximity of the S_1_ minimum. Thus, the induced coherent vibrational dynamics facilitates efficient ISC, which effectively competes with the ultrafast IC.

### 2.5 Insights on the Deactivation Mechanism

We now examine the mechanistic details of the deactivation of photoexcited AcAc and the role of the triplet manifold. Figure 4(a) shows the S_1_ and S_2_ potential energy surfaces in the space spanned by the ESIHT coordinate coupled to BLA (ϕ), which drives the S_2_ → S_1_ IC and ring opening (φ), which guides the system toward the S_1_ minimum.

Starting from the asymmetric geometry of the S_0_ minimum (with respect to the O6–H–O10 distances, see atom labels in Figure 4(b)), photoexcitation to S_2_(ππ*) triggers coherent coupled ESIHT/BLA toward a symmetric geometry in which the hydrogen is equally distant from the two oxygens (see Figures 4(a) and S11). Along this path, notably well before the symmetric geometry is reached, S_2_ crosses with S_1_ and they exchange their electronic nature. As a result, S_1_ acquires ππ* character in the vicinity of the symmetric geometry (see Figure S11). The symmetric geometry represents, however, an unstable point on S_1_, as the ring-opening mode (mode 14–885 cm^–1^) drives the system toward one of the two identical nπ* minima characterized by asymmetric O6–H–O10 distances (Figure S5). Despite the fact that further hydrogen transfer is not favored after the decay to S_1_, the momentum gained along the ESIHT coordinate allows a significant portion of the population (33% of trajectories) to complete the process compared to 67% of trajectories that undergo an interrupted ESIHT.

Figure 4(b) shows the potential energy profiles along the full deactivation path (obtained through geodesic interpolation) connecting the FC point (S_0_ min) to the S_1_/S_0_ MECI through the S_2_/S_1_ MECI and S_1_ minimum. Our simulations show a branching of the population on S_1_: most trajectories (45%) proceed through the S_1_ → S_0_ nonadiabatic decay without the involvement of triplets, about 16% of the population undergoes ISC, whereas about 39% of trajectories remain trapped around the S_1_ minimum for the entire duration of the simulation.

In detail, all the trajectories that reach the ground state relax in the vicinity of the same S_1_/S_0_ MECI. This crossing point is characterized by a high out-of-plane deformation with broken conjugation and exhibits C–O bonds nearly perpendicular to each other, with a significant pyramidalization of the C9 atom[Fn fn1].^21–23^ As AcAc distorts away from the planar S_1_ min geometry toward the S_1_/S_0_ MECI, the nature of the electronic state progressively changes from nπ* to ππ* (see Table S3). This interaction results in an energy barrier of 0.17 eV (S_1_ TS, see Figure 4(b)). The barrier is too low to effectively trap the majority of the electronic population; however, it hinders the ballistic S_2_ → S_1_ → S_0_ decay and slows down the IC to the ps time scale. More refined calculations (see Section S7 of the Supporting Information) suggest that the barrier on S_1_ is probably slightly underestimated in our calculations, rationalizing the mismatch between the experiment and simulation regarding the decay rate of the S_1_ fingerprint (A_2_, respectively, 1.6 and 0.9 ps, see Figure 2(b) and Section S2 of Supporting Information) and leading to an overestimation of the role of the IC mechanism in the simulations.

Concerning the ISC mechanism, we observe that S_1_(nπ*) is nearly degenerate with two triplet states (T_1_(nπ*) and T_2_(ππ*)) in the vicinity of the S_1_ minimum, being effectively coupled to T_2_ in agreement with El-Sayed’s rule. As elucidated in Section 2.4, vibrational dynamics around S_1_ min facilitate the S_1_ → T_2_ ISC. This triplet state acts as a doorway, which rapidly funnels the population to T_1_ (see the inset of Figure 4(b)). Upon T_2_ → T_1_ IC, the ππ* nature is retained and becomes the leading configuration of T_1_. Opposite to what is observed for the singlet manifold (S_1_), the out-of-plane deformation is not associated with overcoming a barrier on T_1_ (Figure 4(b)). In fact, the T_1_ minimum is twisted. This rationalizes the observed shift in the tr-PES signal as molecules in the singlet manifold remain trapped around the planar geometry and give rise to a constant signal, whereas the molecules in the triplet manifold relax to the twisted geometry (T_1_ min), blue-shifting the PES signal.

Eventually, we note that our perturbatively corrected multiconfigurational (CASPT2) simulations predict a different singlet/triplet branching ratio with respect to previous studies performed at the multiconfigurational CASSCF level,^17^ which predicted a significantly faster ISC rate. For a better comparison, we have performed a full characterization at the CASSCF level (see Section S8 of the Supporting Information) finding out several artifacts. In fact, CASSCF strongly underestimates the S_1_ → S_0_ decay rate and the T_1_–S_1_ energy gap, thus strongly favoring the ISC channel.

## 3 Conclusions

In conclusion, we have shown how the combination of sub-20 fs tr-PES and high-level simulations allows us to disentangle the ultrafast and complex dynamics of photoexcited AcAc with an unprecedented level of detail. The excellent agreement of simulations and experiments allows us to identify the key steps in the photoinduced process. The primary steps of the AcAc relaxation mechanism have been experimentally and theoretically investigated, highlighting the crucial role of the ultrafast ESIHT process that initiates the dynamics. We were able to experimentally resolve for the first time the ultrafast decay of the spectroscopic feature of S_2_ and the concurrent rise of the S_1_ signal (respectively, A_1_ and A_2_ in Figure 2(a) and (b)). The ultrafast deactivation of the bright S_2_ state is funneled by the presence of a S_2_/S_1_ crossing near the Franck–Condon point, which is reached mainly through ESIHT and C–C/C–O BLA coordinates activated immediately after photoexcitation. These motions are resolved in the coherent mode analysis (modes highlighted in green in Figure 3). Subsequently, the system coherently evolves toward the S_1_ minimum along the vibrational modes associated with deformations that take the molecule to a ring-opened configuration (modes highlighted in orange in Figure 3). The presence of a barrier on S_1_ allows part of the population to oscillate around the S_1_ minimum and undergo ISC to the triplet states. The fast ISC process suggested by previous studies^16–18^ is confirmed by our simulations, with about 16% of the population, which is already in T_1_ after only 700 fs. Experimentally, the transfer of population to T_1_ causes a shift of the signal toward larger binding energy, which is unambiguously linked to a characteristic signal centered at 6.5 eV with a rising time of 1600 fs (A_3_ in Figure 2(a) and (b)). The experimental and theoretical results reported provide a significant step forward in the understanding of the ultrafast nonadiabatic dynamics of acetylacetone, showing the importance of an extreme temporal resolution to disentangle such complex relaxation mechanism.

## 4 Methods

### 4.1 Experiments

The experimental setup is described in ref 20 and in the Supporting Information. Briefly, a 500-nJ UV pump pulse, with a central wavelength of 268 nm and a bandwidth of 15 nm, was used to resonantly excite the ππ* state of AcAc (Figure S1b). XUV probe pulses were obtained by a high-order harmonic (HH) generation source equipped with a time-delay compensated monochromator that allows spectral selection while preserving the time duration of the harmonics.^24,25^ For the present experiments, HH15, with a central photon energy of 24.7 eV and a bandwidth of 300 meV, was selected (Figure S1a). The pump and probe pulses were recombined in the interaction region in a noncollinear configuration, where AcAc (Merck, 99%) at room temperature was delivered through an effusive source. The generated photoelectrons were collected in a time-of-flight spectrometer. A sub-20 fs instrumental response function was obtained through an XUV-UV photoelectron in situ cross-correlation in argon, shown in Figure S1 of the Supporting Information.

### 4.2 Simulations

#### 4.2.1 Electronic Structure

The ground-state geometry of enolic acetylacetone was optimized at the MP2/cc-pVDZ level of theory in the gas phase. All of the excited state minima and transition states (S_1_ min, T_1_ min, S_1_ TS) as well as minimum-energy conical intersections (S_2_/S_1_ MECI, S_1_/S_0_ MECI, T_2_/T_1_ MECI) were optimized at the XMS-CASPT2/CASSCF­(10,8)/cc-pVDZ level of theory, including five electronic states in the state averaging procedure for singlets and two for triplets, respectively (see structures and active orbitals in Figures S5–6). For all minima and transition states, the final geometry was validated by a frequency calculation at the corresponding level of theory. The minimum-energy path between key geometries was approximated by geodesic interpolation.^26^

#### 4.2.2 Molecular Dynamics

We run 103 Tully’s fewest switches surface hopping trajectories sampling initial conditions from Wigner distribution at 300 K (excluding low-frequency CH_3_ torsional modes and high-frequency C–H stretching modes). After sampling 600 frames (geometries + momenta) by means of Wigner sampling (at 300 K), we have calculated the valence electronic structure for each one of them. To reproduce the experimental conditions, only frames showing a bright excited state with excitation energy under the envelope of the experimental excitation pulse were selected for running dynamics simulations (103 samples). All trajectories were started on the bright ππ* state (S_2_) and propagated at the XMS-CASPT2/CASSCF­(10,8)/cc-pVDZ level of theory for 700 fs with a time step of 0.5 fs, applying decoherence^27^ and rescaling the velocity along a nonadiabatic coupling (NAC) vector after a hopping event to conserve total energy. To model IC between states of the same spin multiplicity, we employed time-derivative couplings^28^ for the propagation of electronic-state amplitudes. Instead, ISC was modeled with the spin-diabatic approach described by Cui and Thiel^29^ and by approximating the S_X_–T_Y_ spin–orbit coupling as the sum of the moduli of the couplings associated with all spin sublevels (*m*_S_ = 1, 0, −1) of a given triplet state Y. The statistical error on the population dynamics introduced by finite sampling was estimated by bootstrap analysis^30,31^ (see Supporting Information). All calculations (static and dynamics) were performed with the software COBRAMM^32–34^ interfaced with OpenMolcas^35^ for the electronic structure calculations and Gaussian^36^ for the optimizer.

#### 4.2.3 Spectroscopy Simulation

For the simulation of tr-PES spectra, we have employed the pump–probe simulation utility available with COBRAMM:^33,37^ we have calculated the energies of the lowest 6 ionized states (XMS-CASPT2/SA6-CASSCF­(9,8)/cc-pVDZ) at intervals of 5 fs along each trajectory, together with the Dyson intensities of the transitions associated with the active state at each time. In this way, the electron binding energy is sampled across a wide range of geometries, accounting for the modulation of the signal that could arise from differences in the topologies of the potential energy surfaces of the valence/ionized states. This data was convoluted in energy and time to obtain the simulated tr-PES spectrum of acetylacetone in the gas phase (broadening parameters: time fwhm = 10 fs, energy fwhm = 0.15 eV).

## Supplementary Material



## Data Availability

The experimental data (time-resolved photoelectron spectra), the results of the simulations (simulated photoelectron spectra and populations), and the results of the data analysis (center of mass, global fit, Fourier transform and Gabor transform) are available via Zenodo. Code availability: COBRAMM is available on GitLab at https://gitlab.com/cobrammgroup/cobramm. OpenMOLCAS (v. 23.10) is available at https://gitlab.com/Molcas/OpenMolcas.git. Gaussian (v. 16) is available from Gaussian Inc., subject to licensing restrictions.

## Footnotes

a The degree of out-of-plane deformation and conjugation distortion are quantified by the Tw and C7–C9 conjugation parameters whose definition is provided in Figure 4, and further details are in the Supporting Information.

## Notes

Experimental details, global fit analysis, optimized geometries, additional computational details, contributions to the total tr-PES signal, diabatic states along the HT coordinate, refinement of the S_1_ barrier, comparison with CASSCF simulations, and Cartesian geometries (PDF)
