# Supplementary material for: Mapping Excited-State Decay Mechanisms in Acetylacetone by Sub-20 fs Time-Resolved Photoelectron Spectroscopy
Source: J Am Chem Soc. 2025 Aug 13;147(34):30785–93. doi: 10.1021/jacs.5c06327 (PMC12395474; doi:10.1021/jacs.5c06327)
Supplement: Supplementary file 1 [file ja5c06327_si_001.pdf]

Mapping Excited-state Decay Mechanisms in Acetylacetone by Sub-20 fs Time-resolved Photoelectron Spectroscopy

Supporting Information

Stefano Severino[1†], Flavia Aleotti[2†], Lorenzo Mai[1], Aurora Crego[3,4], Fabio Medeghini[1], Fabio Frassetto[5], Luca Poletto[5], Matteo Lucchini[1,3], Francesco Segatta[2], Maurizio Reduzzi[1], Mauro Nisoli[1,3], Artur Nenov[2*], Rocío Borrego-Varillas[3*]

[1]Dipartimento di Fisica, Politecnico di Milano, Piazza Leonardo da Vinci 32, Milano, 20133, Italy.

[2]Department of Industrial Chemistry "Toso Montanari", University of Bologna, Via Piero Gobetti, 86, Bologna, 40129, Italy, Italy.

[3]Institute of Photonics and Nanotechnologies, CNR (CNR-IFN), Piazza Leonardo da Vinci 32, Milano, 20133, Italy.

[4]Grupo de Investigación en Aplicaciones del Láser y Fotónica, Departamento de Física Aplicada, Universidad de Salamanca, Salamanca, E-37008, Spain.

[5]Institute for Photonics and Nanotechnologies, IFN-CNR, via Trasea 7, Padova, 35131, Italy.

*Corresponding author(s). E-mail(s): artur.nenov@unibo.it; rocio.borregovarillas@cnr.it;
Contributing authors: stefano.severino@polimi.it; flavia.aleotti@unibo.it;
†These authors contributed equally to this work.

Contents

1 Experimental details 2

2 Global Fit Analysis **3**

3 Optimized geometries **7**

4 Additional computational details **8**
4.1 Active orbitals 8
4.2 Excited state frequencies 9
4.3 Energetics and nature of the neutral electronic states 11
4.4 Binding energies at relevant stationary points 11
4.5 Bootstrap analysis 12

5 Contributions to the total tr-PES signal **15**

6 Diabatic states along HT coordinate **15**

7 Refinement of S_1 barrier **16**

8 Comparison with CASSCF simulations **18**

9 Cartesian geometries **21**
9.1 CASPT2 optimized geometries 21
9.2 CASSCF optimized geometries 24

1 Experimental details

The pump pulse is spectrally centered at 267 nm, while for the probe pulse we selected a portion of the 15th harmonic of the 800 nm pulse driving the high harmonic generation (see Figure S1(a) and (b)). The sub-20-fs Instrument Response Function (IRF) was obtained from a cross-correlation signal in argon shown in Figure S1(c). The pump and probe $1/e^2$ beam waists were estimated to be 150 μm and 90 μm, respectively. The pump pulse energy for the main experimental data set shown in Figure 1(a) was set to 500 nJ.

The sample, which is found in liquid phase at room temperature and atmospheric pressure, was bought from Merck. This was delivered to the experimental chamber through an effusive needle of 0.5 mm aperture. The main data set shown in Figure 1 of the main paper is the average of 8 consecutive measurements performed with 4 fs step size over a range of over 700 fs. Every 5 delays, a background photoelectron spectrum was acquired blocking the pump arm. The error on each pixel of the 2D map was obtained as the standard deviation of the 8 scans (see error bars on the lineouts in Fig. S3(c)). The repeatability of the measurement was tested performing different scans with different configurations. In Figure S2, for example, we show the dataset acquired with 39 eV probing energy with 5 fs, 10 fs, and 50 fs step size, respectively in (a), (b) and (c). All measurements were taken on different days to test the reproducibility of the data. Note that the same spectroscopic features as in Figure 1 are obtained, e.g. the sub-50-fs binding energy shift from 4.5 eV to 5.75 eV and the periodic chemical shifts. In particular, the dataset in (c) has been used to confirm the long timescales decay constants, i.e. τ_{d_2} and τ_{r_3} from the global fit analysis (see next

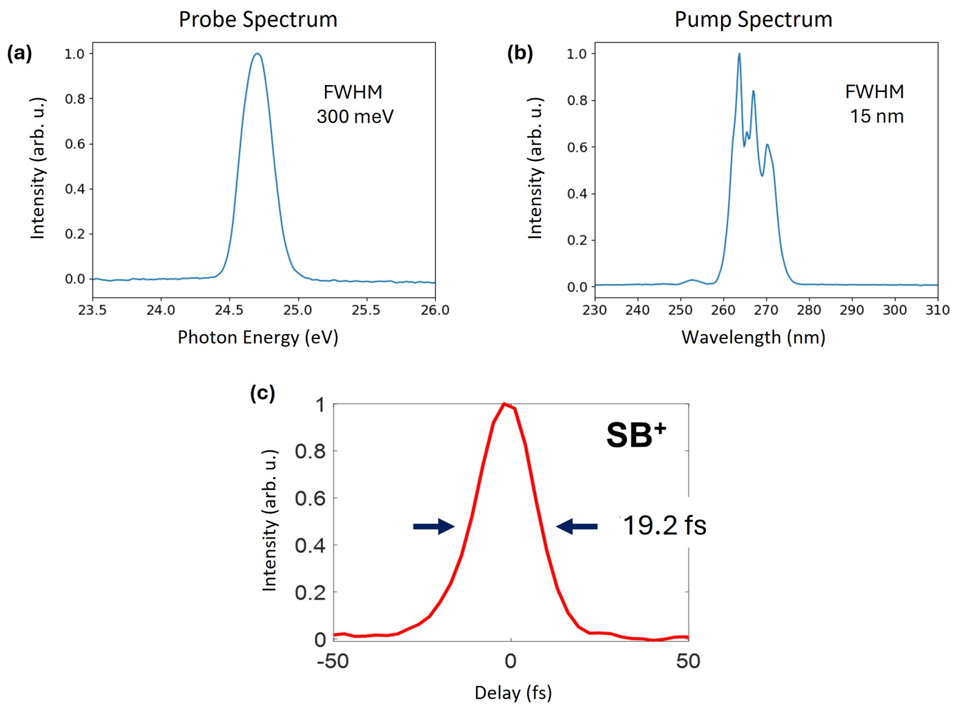

Fig. S1: **Pump and probe characterization.** (a) and (b) show, respectively, the probe and pump spectra used for the measurement; (c) integration of the 2D photoelectron trace in Argon over corresponding to the two photons process (XUV+UV) showing an IRF of 19.2 fs.

section). The centre of mass plotted in Figures 1 and 2 are obtained by performing a weighted average of the binding energy as function of the pump-probe delay, in which the weights are given by the intensities of the signal at each pixel. The result is a quantity that enables following the evolution of the average binding energy of the excited wavepackets as function of the delay. The CM and its error bars (see Fig. 2(d) and 3(a)) are obtained calculating the CM for each of the 8 independent scans and then performing their average and standard deviation. Similarly, we obtained the Fourier Transform analysis and its standard deviations in Fig. 3(b) performing the analysis on each of 8 independent measurements.

2 Global Fit Analysis

With the aim of disentangling the independent components characterizing the tr-PES in Figure 1(a), we apply a least square fitting model to the tr-PES(E, τ), where E is

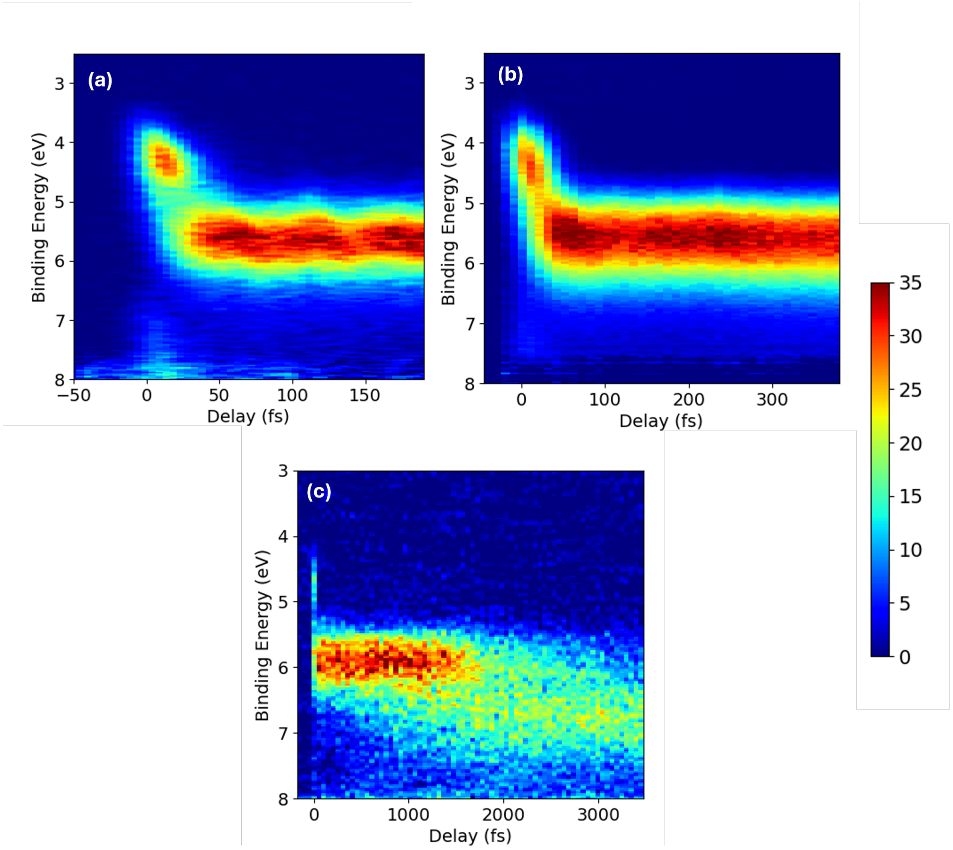

Fig. S2: **tr-PES in AcetylAcetone with 39 eV probing energy.** Scans performed with 39 eV probing energy with step sizes of (a) 5 fs (for pump-probe delays up to 200 fs), (b) 10 fs (for pump-probe delays up to 400 fs) and 50 fs (for pump-probe delays up to 3.5 ps).

the photon energy and τ is the pump-probe delay as

$$PES(E,\tau) = \sum_{i=1}^{3} A_i(E) f_i(\tau)$$

The first component is modeled as:

$$A_1(E) f_1(\tau)$$

$$f_1(\tau) = \frac{1}{\gamma\sqrt{2\pi}} e^{-\frac{1}{2}(\frac{\tau}{\gamma})^2} * e^{-\frac{\tau-\tau_0}{\tau_{d_1}}}$$

where $A_1(E)$ is the spectral amplitude and $f_1(\tau)$ is a function comprised of a convolution, in time, of a Gaussian and an exponential decay functions. This component is

modeled to reproduce the signal centered around 4.5 eV, whose rise is defined by the pump-induced electronic excitation. The Gaussian function represents the instrument response function and is characterized by its τ_0 and γ parameters. A_2 and A_3 are, instead, modeled as an exponential rise function plus an exponential decay function, convoluted with a Gaussian function with the same τ_0 and γ as f_1:

$$A_i(E)f_i(\tau)$$

$$f_i(\tau) = \frac{1}{\gamma\sqrt{2\pi}}e^{-\frac{1}{2}(\frac{\tau}{\gamma})^2} * e^{-\frac{\tau-\tau_0}{\tau_{d_i}}}(1 - e^{-\frac{\tau-\tau_0}{\tau_{r_i}}}) \qquad i = 2, 3$$

The second and third components are modeled to reproduce, respectively, the signals centered at 5.75 eV and 6.5 eV. τ_{r_2} has been constrained to be equal to τ_{d_1}, assuming a direct flow of population from the first to the second component. In contrast, τ_{r_3} has been left free in order to verify the existence of a channel different from the direct flow of population from the second to the third component. In Figure S3 we show the comparison between the experimental data and the result of the fitting procedure. The fitting results and their errors are obtained, respectively, as mean and standard deviation of the results of the global fit analysis performed on each of the eight

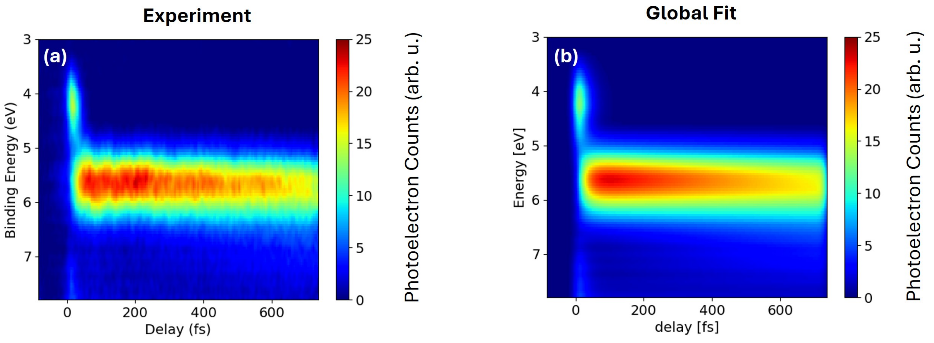

i	γ	τ_0	τ_r	τ_d
1	9±1	1±1		23±2
2		1±1	23±2	1520±135
3		1±1	1610±125	∞

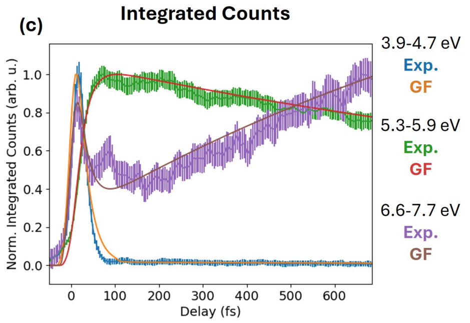

Fig. S3: **Global Fit Analysis of the experimental data.** The global fit routine described in the text has been performed on the experimental tr-PES in (a) and its result is shown in (b). Panel (c) shows the comparison between the integrated photoelectron counts of the experimental trace and the global fit result. The table shows the parameters obtained from the fitting procedure expressed in femtoseconds.

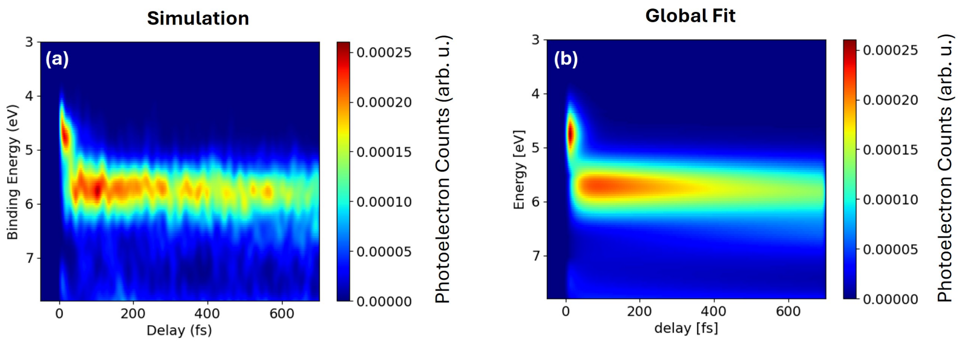

i	γ	τ_0	τ_r	τ_d
1	5	5		20
2		5	20	903
3		5	1653	∞

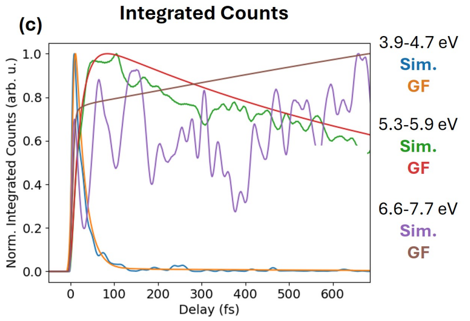

Fig. S4: **Global Fit Analysis of the simulated data.** The global fit routine described in the text has been performed on the simulated tr-PES in (a) and its result is shown in (b). Panel (c) shows the comparison between the integrated photoelectron counts of the experimental trace and the global fit result. The table shows the parameters obtained from the fitting procedure expressed in femtoseconds.

measured scans. A_i and f_i are presented in Figure 2(c) and (d) of the main paper. For better comparison,s in Figure S3 (c) we present the integrated photoelectron counts in the relevant regions of the experimental trace, compared to the ones of the global fit trace in Figure S3(b). Similarly, in Figure S4 are presented the results of the global fitting procedure performed on the simulated tr-PES. Also in this case, A_i and f_i are presented in Figure 2(c) and (d) of the main paper (dotted lines).

Comparing the results of the fitting procedures on the simulated and experimental traces, we observe that:

1) τ_{d_1} values are comparable, confirming the ultrafast decay from the first surface.

2) from the experimental data, τ_{d_2} and τ_{r_3} are statistically the same, confirming the direct flow from the second to the third component as the main channel of relaxation; on the contrary, the simulated data show significantly different values for τ_{d_2} and τ_{r_3}. This will be discussed in detail in section 7;

3) the fitted value of τ_{r_3} matches very well between theory and experiment and is linked to the ultrafast intersystem crossing.

3 Optimized geometries

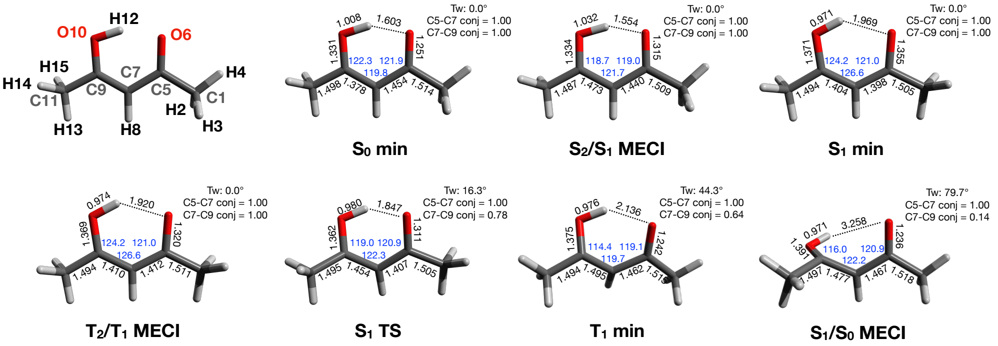

Fig. S5: Optimized structures (MP2/cc-PVDZ for S_0 min, XMS-CASPT2/CASSCF(10e,8o)/cc-pVDZ for the rest, with state averaging on 5 roots for singlets and on 2 roots for triplets). Bond lengths are reported in black. Relevant ring angles are reported in blue. Tw = O10-C9-C5-O6 dihedral. Conjugation between C5-C7 and C7-C9 carbon atoms is measured as the cosine of the angle between the corresponding p orbitals (1 = complete conjugation, 0 = orthogonal orbitals, no conjugation).

To measure the degree of π-conjugation between the C5-C7-C9 atoms (see Figure S5) we have considered for each of them the plane defined by the three atoms to which it is connected, and we have calculated the normal vector to such plane (defining the direction of the corresponding p orbital). The cosine of the angle between C5-C7 p orbitals and C7-C9 p orbitals will measure the degree of π-conjugation. With this parameter, we get 1 for perfect conjugation and 0 for orthogonal p orbitals (no conjugation). The pyramidalization of C9 (bearing the -OH substituent) that is progressively increasing along S_1 TS $\rightarrow$ T_1 min $\rightarrow$ S_1/S_0 MECI breaks the C7-C9 π-conjugation, as reflected in the concurrent decrease in our parameter.

4 Additional computational details

4.1 Active orbitals

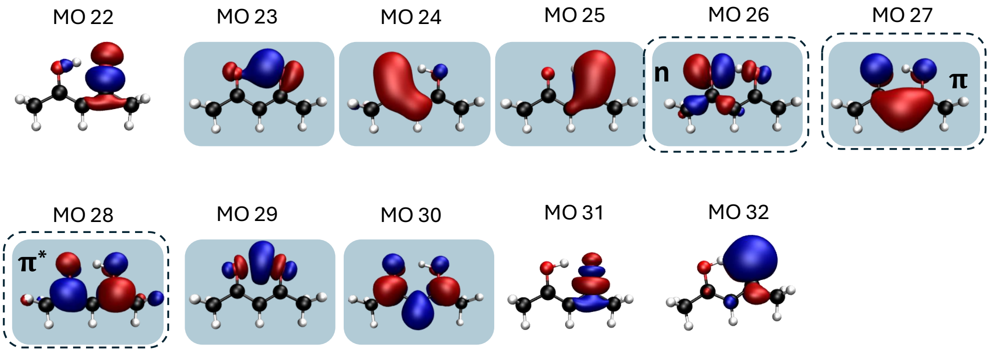

Fig. S6: AcAc active space orbitals (CASSCF/cc-pVDZ). The shaded background indicates orbitals selected for the final (10,8) active space, while orbitals involved in the nπ^* and $\pi\pi^*$ excitations (dominant configurations in S_1 and S_2 states, respectively) are further highlighted by dashed boxes.

The excited state energy convergence with respect to active space size was assessed (Table S1), and the (10,8) active space (shaded orbitals in Figure S6) was eventually selected to balance between accuracy and affordable computational cost of XMS-CASPT2/CASSCF (to be used for dynamics).

Table S1: Convergence of excited state energies (XMS-CASPT2/CASSCF/cc-pVDZ, relative to S_0 at the corresponding level of theory) with active space size. In the last row, the experimental UV-Vis absorption maximum[1] is reported for comparison. (AS (8,8) included MOs 24, 25, 26, 27, 28, 29, 30, 32; AS (12,10) included MOs 22, 23, 24, 25, 26, 27, 28, 29, 30, 31, see Figure S6)

**Active space size**	**S_1 (eV)**	**S_2 (eV)**
(8,8)	4.39	4.64
(10,8)	4.41	4.69
(12,10)	4.31	4.66
Exp.	-	4.68

4.2 Excited state frequencies

In order to explain the frequency peaks obtained from Fourier analysis (of experimental and simulated spectra) in terms of excited states modes, we have performed an optimization and frequency calculation on S_1 (XMS-CASPT2/SA5-CASSCF(10,8)/cc-pVDZ). Then, we have projected the gradients of S_1 ($n\pi^*$) and S_2 ($\pi\pi^*$) calculated at the Franck-Condon point on the resulting normal modes, in order to identify the most activated modes on each excited state. In the case of S_2 it was not possible to converge a geometry optimization, due to the immediate crossing with S_1. Therefore, we assumed the S_2 normal modes and frequencies to be similar to those of S_1, and we have projected the S_2 gradient on S_1 normal modes. We identified some normal modes that match the peaks in the FT of the experimental and simulated traces (reported in the main text). Accordingly, the same modes showed large contributions to the S_2 and/or S_1 gradients (measured through the associated reorganization energy, see Table S2) The most contributing mode to both S_2 and S_1 gradients at the FC point is mode 2 (159 cm^{-1}). This low-frequency mode is outside the FT window, and the associated peak is not revealed. However, it mostly represents the ring opening/closing coordinate (Figure S7) leading to S_2/S_1 MECI (ring closure, O-H shorter distances) and S_1 minimum (ring opening, asymmetric OH distances).

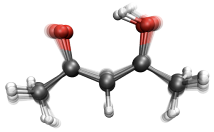

Fig. S7: S_1 normal mode 2 (159 cm $^{-1}$).

Table S2: Normal modes associated with FT peaks and their contribution to S_2 or S_1 gradients (reorganization energy, eV)

Mode n°	Frequency (cm^{-1})	Reorg. en. (eV)
S_2 active modes		
2^1	159	0.35
11	589	0.04
21	1212	0.04
25	1419	0.05
31	1539	0.04
S_1 active modes		
2^1	159	0.23
14	885	0.09
20	1130	0.09
22	1334	0.15
25	1419	0.10

[1]Mode 2 is outside the FT window (low frequency mode), but its contribution is reported in this table as it is the largest on both excited states.

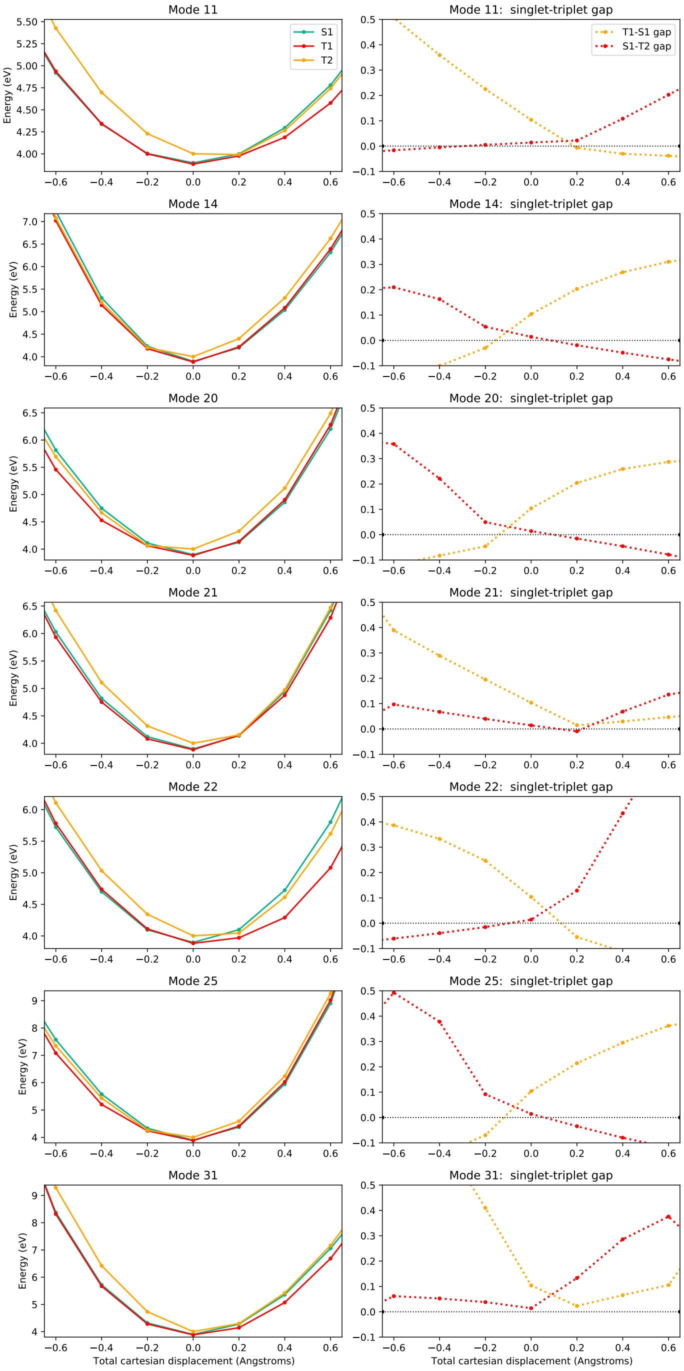

Fig. S8: Left column: potential energy profiles of S_1 (green), T_1 (red) and T_2 (orange) for displacements along the excited state normal modes activated on S_2 (modes 11, 21, 25, 31) and S_1 (modes 14, 20, 22, 25). Right column: modulation of S_1-T_1 and S_1-T_2 gap along the same modes.

4.3 Energetics and nature of the neutral electronic states

Table S3: Excited state energies (eV) and electronic nature (dominant electronic configurations, weight in parentheses. HF = closed shell.) at relevant optimized geometries (XMS-CASPT2/CASSCF(10,8)/cc-pVDZ; state averaging on 5 roots for singlet states, 2 roots for triplet states). The energy of the ground state minimum (S_0 min) is used as reference for calculating the relative energies ΔE.

	S_0		S_1		S_2		T_1		T_2	
	ΔE	nature	ΔE	nature	ΔE	nature	ΔE	nature	ΔE	nature
S_0 min	0.00	HF (0.84)	4.41	$n\pi^*$ (0.88)	4.69	$\pi\pi^*$ (0.69)	3.86	$\pi\pi^*$ (0.85)	4.24	$n\pi^*$ (0.88)
S_2/S_1 MECI	0.26	HF (0.83)	4.40	$n\pi^*$ (0.87)	4.40	$\pi\pi^*$ (0.69)	3.68	$\pi\pi^*$ (0.85)	4.29	$n\pi^*$ (0.88)
S_1 min	0.59	HF (0.80)	3.90	$n\pi^*$ (0.88)	4.88	$\pi\pi^*$ (0.67)	3.88	$n\pi^*$ (0.91)	4.00	$\pi\pi^*$ (0.90)
T_2/T_1 MECI	0.39	HF (0.81)	3.93	$n\pi^*$ (0.88)	4.77	$\pi\pi^*$ (0.67)	3.88	$n\pi^*$ (0.84)	3.88	$\pi\pi^*$ (0.82)
S_1 TS	1.17	HF (0.79)	4.07	$n\pi^*$ (0.42) $\pi\pi^*$ (0.40)	4.72	$n\pi^*$ (0.28) $\pi\pi^*$ (0.44)	3.73	$\pi\pi^*$ (0.82)	4.30	$n\pi^*$ (0.85)
T_1 min	2.21	HF (0.68) $\pi\pi^*$ (0.20)	4.09	HF (0.16) $\pi\pi^*$ (0.59)	5.11	$n\pi^*$ (0.82)	3.46	$\pi\pi^*$ (0.86)	5.00	$n\pi^*$ (0.88)
S_1/S_0 MECI	3.40	$\pi\pi^*$ (0.77)	3.40	HF (0.75)	4.99	$n\pi^*$ (0.85)	3.49	$\pi\pi^*$ (0.86)	4.96	$n\pi^*$ (0.90)

4.4 Binding energies at relevant stationary points

Table S4: Binding energy (BE, in eV) from the photoactive states $S_0/1/2$ and T_1 at relevant stationary points (S_0 min, S_1 min, T_1 min) computed at the XMS-CASPT2/CASSCF(10,8)/cc-pVDZ level of theory; state averaging on 5 roots for singlet states, 2 roots for triplet states). Dyson intensities reported in parentheses. BE giving rise to tr-PES signals in the tr-PES probe window are highlighted in boldface.

	S_0		S_1		S_2		T_1	
	ionized state	BE	ionized state	BE	ionized state	BE	ionized state	BE
S_0 min	D_1	8.92 (0.89)	D_2	4.99 (0.44)	D_1	**4.16 (0.45)**	D_1	5.02 (0.82)
	D_2	9.47 (0.83)	D_5	8.78 (0.62)	D_3	7.47 (0.15)	D_3	8.28 (0.16)
	D_3	12.19 (0.71)	D_6	9.35 (0.25)	D_4	8.49 (0.12)	D_5	9.36 (0.28)
	D_4	13.25 (0.65)			D_6	9.08 (0.67)		
S_1 min	D_1	7.35 (0.86)	D_1	**5.20 (0.44)**	D_2	2.25 (0.47)	D_1	5.61 (0.88)
	D_2	7.50 (0.77)	D_4	8.64 (0.66)	D_3	4.58 (0.13)	D_4	9.18 (0.12)
	D_3	9.68 (0.64)	D_6	10.44 (0.21)	D_5	6.87 (0.15)	D_6	9.88 (0.51)
	D_5	11.96 (0.65)			D_6	7.65 (0.53)		
T_1 min	D_1	7.70 (0.83)	D_1	5.83 (0.52)	D_2	5.89 (0.43)	D_1	**6.45 (0.89)**
	D_2	8.79 (0.65)	D_2	6.92 (0.11)	D_4	7.87 (0.82)	D_3	9.12 (0.82)
	D_5	11.03 (0.10)	D_3	8.50 (0.36)			D_4	9.52 (0.35)
	D_6	11.72 (0.61)	D_4	8.90 (0.20)			D_5	9.78 (0.15)
			D_5	9.16 (0.41)				

4.5 Bootstrap analysis

We have performed bootstrap analysis to estimate the statistical error on the population dynamics due to finite sampling of initial conditions.[2] Specifically, we have performed a stratified sampling bootstrap analysis[3], in which the original ensemble is divided into clusters (i.e., decay paths in our case) and bootstrapping is performed with the constraint to keep a fixed ratio of trajectories for each cluster. The analysis was performed through the following steps:

1. Estimate the decay/rise times of the electronic populations using the original dataset (103 trajectories);
2. Create three clusters representing the three decay paths based on the active state at the end of the simulation time (cluster 1 = 46 trajs ending on S_0, cluster 2 = 40 trajs ending on S_1, cluster 3 = 17 trajs ending on T_1);
3. Create a large number of replica ensembles (of 103 trajectories each), by random sampling (with replacement) from each cluster;
4. Estimate decay/rise times for each replica;
5. Obtain mean and standard deviation of decay/rise times.

The S_2, S_1 and T_1 electronic state populations were fitted using the following functions:

- S_2: monoexponential decay

$$f_{S_2}(t) = A_d \exp\left(-\frac{t}{\tau_d}\right)$$

- S_1: monoexponential rise + biexponential decay

$$f_{S_1}(t) = \left[1 - \exp\left(-\frac{t}{\tau_r}\right)\right]\left[A_{d1}\exp\left(-\frac{t}{\tau_{d1}}\right) + A_{d2}\exp\left(-\frac{t}{\tau_{d2}}\right)\right]$$

- T_1: monoexponential rise

$$f_{T_1}(t) = A_r\left[1 - \exp\left(-\frac{t}{\tau_r}\right)\right]$$

the fitted parameters (from original data and bootstrap analysis) are reported in Table S5.

The fitted functions nicely reproduce the population dynamics from the original dataset (Figure S9). Comparing with the decay times associated to the experimental/simulated signals we not that:

- the decay time of S_2 (8 fs) is a shorter than the decay time associated to the A_1 spectral component (23 ± 2 fs, see main text). This is due to the fact that the $\pi\pi^*$ character is retained for some time after the $S_2 \rightarrow S_1$ hop;
- S_1 population is better fitted using a biexponential decay, in contrast to the monoexponential decay used in the global fit analysis for the A_2 component. The short

component (77 fs) reflects the ultrafast $S_1 \rightarrow S_0$ decay, which however is overestimated by our simulations as a consequence of barrier underestimation (see section S7). On the other hand, the longer component (1195 fs) is in line with the lifetime of the A_2 signal from experiments (1520 ± 135 fs);

- The T_2 rise time (656 fs) is shorter than that of the A_3 spectral component (1610 ± 125). This is most likely due to the fact that the PES signal from T_1 initially overlaps with that of S_1;
- Due to limited number of samples reaching T_1 (cluster 3 in bootstrap analysis), any statistical analysis on its raise time produces no meaningful results (this is reflected in the large standard deviation obtained from bootstrapping, see Table S5)

Table S5: Bootstrapping analysis: average pre-exponential factors and decay times (± standard deviation) obtained using 10000 and 15000 randomly-sampled replicas. (* too few trajectories)

		Original dataset	Bootstrapping copies 10000	15000
S_2	A_d	1.12	1.13 ± 0.05	1.13 ± 0.05
	τ_d (fs)	8	8 ± 1	8 ± 1
S_1	τ_r (fs)	10	11 ± 34	11 ± 3
	A_{d1}	0.71	0.69 ± 0.08	0.69 ± 0.07
	τ_{d1} (fs)	1195	1335 ± 319	1325 ± 305
	A_{d2}	0.4	0.5 ± 0.3	0.5 ± 0.3
	τ_{d2} (fs)	77	76 ± 27	76 ± 27
T_1	A_r	0.2	0.3 ± 0.2*	0.3 ± 0.2*
	τ_r (fs)	656	802 ± 789*	798 ± 768*

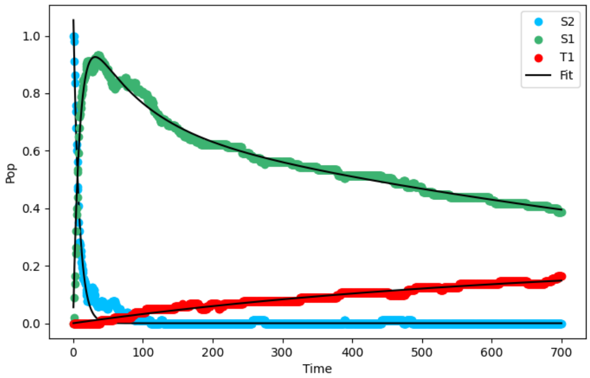

Fig. S9: Results of fitting of the population dynamics from simulations: S_2 (blue, monoexponential decay), S_1 (green, monoexponential rise + biexponential decay) and T_1 (red, monoexponential rise).

5 Contributions to the total tr-PES signal

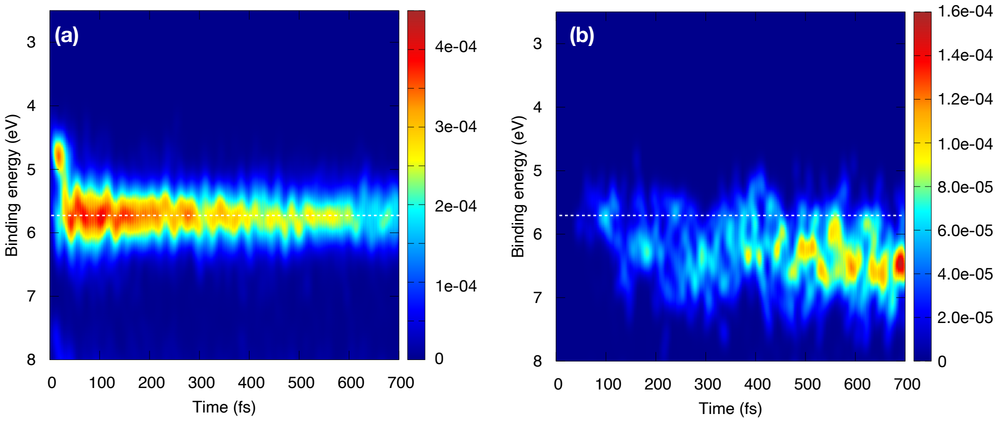

Fig. S10: Contribution of S_1 (a) and T_1 (b) electronic states to the total simulated tr-PES signal reported in this work. The dashed line at 5.75 eV highlights the shift of the T_1 contribution over time, while the signal from S_1 remains constant.

6 Diabatic states along HT coordinate

The potential energy profiles of the S_0, S_1 and S_2 states along the HT coordinate was investigated by means of geodesic interpolation [4] between the two C_s ground state minima. The ground state keeps a closed-shell configuration along all the scan, and shows a transition state of C_{2v} symmetry (Figure S11). In contrast, we observe a clear change in the electronic nature between S_2 and S_1, which allows to draw the profile of the diabatic $\pi\pi^*$ and $n\pi^*$ states reported in Figure S11.

After pump excitation, the bright $\pi\pi^*$ state (S_2) shows a favorable gradient along ESIHT coordinate and starts to evolve towards the symmetric C_{2v} minimum. This destabilizes the $n\pi^*$ state (S_1) and leads to crossing between the two states (before reaching the symmetric structure). After internal conversion to S_1, the ESIHT is disfavored due to the predominant $n\pi^*$ character of the latter state, and the vibrational energy is transferred to other modes.

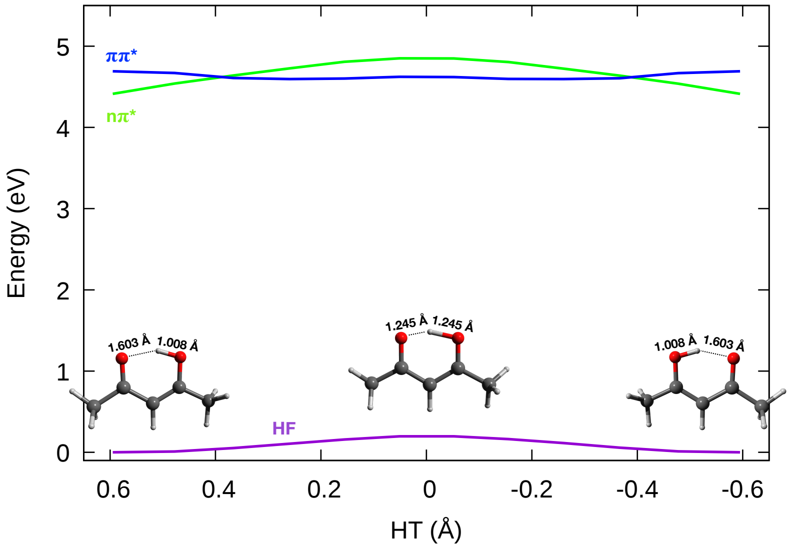

Fig. S11: Diabatic character (level of theory = XMS-CASPT2/SA5-CASSCF(10,8)/cc-pVDZ) of electronic states along the HT coordinate, defined as the difference between the two O-H distances. HF = Hartree-Fock, closed-shell configuration.

7 Refinement of S_1 barrier

To investigate the origin of the mismatch in the time decay constants associated to the experimental ad simulated S_1 signal (BE $\approx$ 5.7 eV) we have performed the optimization of S_1 min and S_1 TS increasing the active space to 12 electrons in 12 orbitals (see Figure S12). In addition, we have also tested the use of a larger basis set (cc-pVTZ). The results are collected in Table S6. For every level of theory, we have confirmed the minima/saddle points nature of S_1 min and S_1 TS by means of frequency calculation.

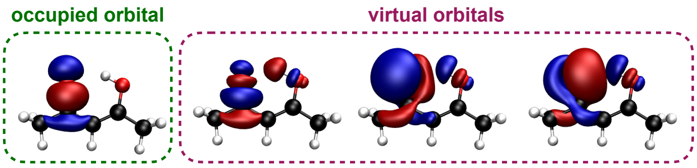

Fig. S12: Active space orbitals added to those of Figure S6 to perform the CAS(12,12) calculations.

Table S6: XMS-CASPT2/CASSCF energies of S_1 min and TS (atomic units, a.u.) and energy barrier (a.u, eV) separating them using different active spaces an basis sets.

Active space	basis set	S_1 min (a.u.)	S_1 TS (a.u.)	Barrier (a.u.)	Barrier (eV)
CAS(10,8)	cc-pVDZ	-344.67507573	-344.66857756	0.00649817	0.177
CAS(12,12)	cc-pVDZ	-344.66935116	-344.66182552	0.00752564	0.205
CAS(12,12)	cc-pVTZ	-345.01536373	-345.00847180	0.00689193	0.189

8 Comparison with CASSCF simulations

The full computational study (static characterization and dynamics simulations) was repeated at CASSCF level using the same active space and basis set (i.e., CASSCF(10,8)/cc-pVDZ) and number of electronic states in state-averaging (5 singlets, 2 triplets) in order to highlight the differences with the CASPT2 results. Table S7 displays the electronic state energies and nature at relevant geometries, while Figure S13 shows the energy profiles along the geodesic interpolation path for S_0 minimum → S_2/S_1 MECI → S_1 minimum → S_1/S_0 MECI. Already from this static characterization we note significant differences with respect to CASPT2 potential energy surfaces:

(a) S_2 excitation energy at FC point is significantly overestimated by CASSCF, and the corresponding gradient towards S_2/S_1 MECI is much steeper. This suggests that CASSCF dynamics will gain large kinetic energy after the $S_2 \rightarrow S_1$ hop;

(b) the relative positions of S_1 and the triplets in the region of S_1 minimum are slightly different between CASSCF and XMS-CASPT2: the former places both T_1 and T_2 below S_1, favoring ISC due to negative energy gap. In contrast, the triplet showing the largest SOC with S_1 at CASPT2 level (T_2) is slightly higher in energy with respect to S_1 at its minimum;

(c) the CASSCF structures of S_1 TS and T_1 minimum are much more distorted (with respect to the planar S_0min, S_2/S_1 MECI and S_1 min) and show a larger degree of C-pyramidalization and deconjugation (see Figure S13). As a consequence, larger distortions are expected at CASSCF level, especially on T_1 PES.

Figure S14 shows the results of CASSCF dynamics. The dynamics was performed using the same propagation setup used for the CASPT2 dynamics, but the initial conditions were sampled using CASSCF S_0 minimum and normal modes. As already predicted by static calculations, CASSCF yields a faster ISC rate and a slower $S_1 \rightarrow S_0$ IC rate compared to CASPT2. The high ISC rate agrees very well with previous CASSCF simulations on the same system[5]. However, this fast increase in triplet

Table S7: Excited state energies (eV) and electronic nature (dominant CI configurations) at relevant optimized geometries (CASSCF(10,8)/cc-pVDZ; state averaging on 5 roots for singlet states, 2 roots for triplet states)

	S_0		S_1		S_2		T_1		T_2	
	E	nature	E	nature	E	nature	E	nature	E	nature
S_0 min	0.00	HF (0.74)<br>$\pi\pi^*$ (0.17)	4.29	nπ^* (0.86)	6.66	HF (0.15)<br>$\pi\pi^*$ (0.59)	3.83	$\pi\pi^*$ (0.81)	4.00	nπ^* (0.85)
S_2/S_1 MECI	0.94	HF (0.86)	4.94	$\pi\pi^*$ (0.75)	4.94	nπ^* (0.84)	4.14	$\pi\pi^*$ (0.90)	4.76	nπ^* (0.87)
S_1 min	0.92	HF (0.63)<br>$\pi\pi^*$ (0.25)	3.42	nπ^* (0.89)	6.43	HF (0.16)<br>$\pi\pi^*$ (0.24)<br>H→L+2 (0.11)<br>H-2→L (0.21)	3.14	nπ^* (0.92)	3.31	$\pi\pi^*$ (0.90)
S_1 TS	3.34	HF (0.12)<br>$\pi\pi^*$ (0.65)	3.91	nπ^* (0.82)	5.75	HF (0.67)	2.86	$\pi\pi^*$ (0.88)	3.45	nπ^* (0.94)
T_1 min	3.02	$\pi\pi^*$ (0.84)	4.73	nπ^* (0.81)	5.33	HF (0.77)	2.84	$\pi\pi^*$ (0.87)	4.46	nπ^* (0.89)
S_1/S_0 MECI	3.49	HF (0.86)	3.49	$\pi\pi^*$ (0.75)	5.68	nπ^* (0.84)	3.30	$\pi\pi^*$ (0.90)	4.08	nπ^* (0.87)

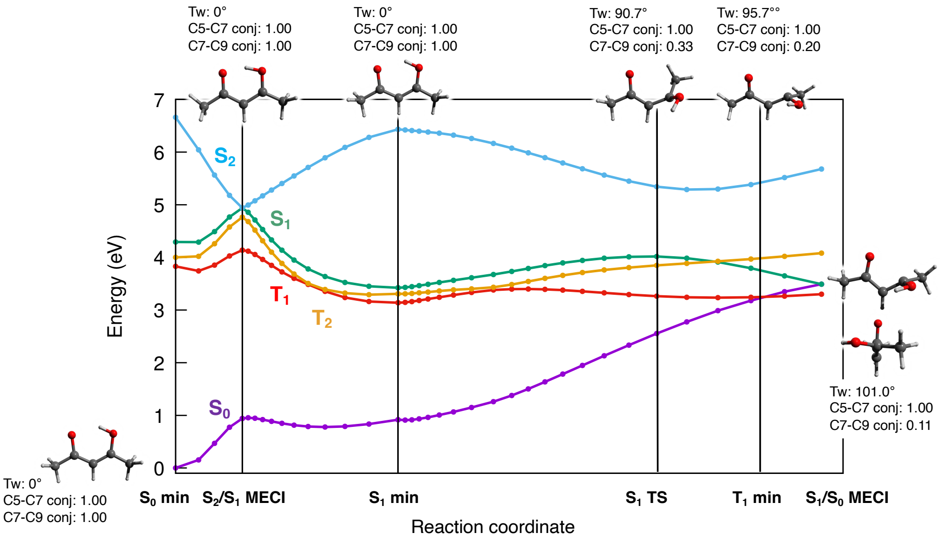

Fig. S13: Potential energy profiles along geodesic interpolation from S_0 min to S_2/S_1 MECI, S_2/S_1 MECI to S_1 min and from S_1 min to S_1/S_0 MECI (via S_1 TS and T_1 min) performed at CASSCF(10e,8o)/cc-pVDZ level (state averaging on 5 roots for singlets and on 2 roots for triplets). Tw = O10-C9-C5-O6 dihedral. Conjugation between C5-C7 and C7-C9 carbon atoms is measured as the cosine of the angle between the corresponding p orbitals (1 = complete conjugation, 0 = orthogonal orbitals, no conjugation).

population is probably an artefact of CASSCF, as already pointed out by the authors of reference [5]. Moreover, the combination of large kinetic energy and large deformations on T_1 (as a consequence of the high S_2 initial energy and distorted T_1 minimum, see before) we observe large amplitude deformations and some fragmentations (especially of the H involved in the ring) in CASSCF dynamics, which were not observed in CASPT2.

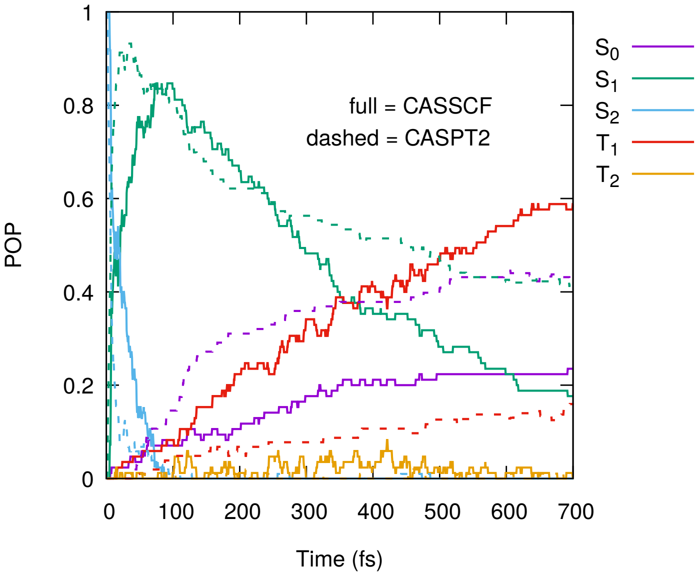

Fig. S14: Population dynamics for CASSCF simulations. The CASPT2 populations are also reported (dotted lines) for comparison.

9 Cartesian geometries

9.1 CASPT2 optimized geometries

S_0 minimum

C	-0.002772	0.000803	0.056126
H	-0.623449	-0.483580	0.828528
H	0.905184	0.382037	0.552864
H	0.273638	-0.732543	-0.713902
C	-0.765030	1.145377	-0.576719
O	-0.986113	1.143568	-1.808490
C	-1.220992	2.224852	0.284654
H	-1.014973	2.202170	1.355972
C	-1.918140	3.283930	-0.255604
O	-2.196804	3.362511	-1.554255
C	-2.430676	4.443432	0.543350
H	-1.791184	2.523692	-1.939043
H	-2.180430	4.340679	1.608476
H	-1.998191	5.379456	0.152945
H	-3.524857	4.514939	0.428228

S_2/S_1 MECI

C	0.020726	-0.040340	0.057650
H	-0.575712	-0.577144	0.817845
H	0.957756	0.290973	0.541383
H	0.272170	-0.742015	-0.750974
C	-0.743107	1.123429	-0.524801
O	-0.992680	1.153013	-1.816058
C	-1.207478	2.206745	0.301633
H	-1.012289	2.204938	1.378261
C	-1.953709	3.344760	-0.261850
O	-2.201054	3.363700	-1.573065
C	-2.462796	4.495973	0.530536
H	-1.770962	2.493179	-1.923884
H	-2.208595	4.386404	1.595364
H	-2.035234	5.446192	0.157818
H	-3.561825	4.581516	0.433274

S_1 minimum

C	0.025520	-0.044073	0.099207
H	-0.006550	0.011036	1.197059
H	1.079057	-0.085264	-0.229179
H	-0.465216	-0.977128	-0.229532
C	-0.670710	1.161689	-0.472444
O	-0.672637	1.165871	-1.827761
C	-1.268805	2.197111	0.252494
H	-1.218695	2.110057	1.341076
C	-1.918744	3.323084	-0.278171
O	-2.061172	3.569902	-1.618938
C	-2.538329	4.396777	0.555947
H	-1.646678	2.851002	-2.122692
H	-2.416242	4.185143	1.628269
H	-2.078622	5.376936	0.333943
H	-3.616968	4.489180	0.333851

S_1 TS

C	1.405927	-2.183287	0.125427
H	1.238514	-2.296873	1.206977
H	2.490103	-2.136128	-0.067372
H	1.008767	-3.065363	-0.405480
C	0.741198	-0.918787	-0.374082
O	0.908976	-0.649526	-1.646319
C	-0.189749	-0.202238	0.399429
H	-0.413460	-0.586449	1.399688
C	-0.951489	0.913405	-0.138586
O	-1.126966	1.003610	-1.486341
C	-1.081752	2.215946	0.583982
H	-0.696306	0.227200	-1.901584
H	-1.146665	2.053866	1.671391
H	-0.205417	2.867356	0.384562
H	-1.981680	2.757268	0.248307

S_1/S_0 MECI

C	-0.047503	-0.012267	0.174157
H	-0.062897	0.053395	1.275026
H	0.937137	-0.362922	-0.163521
H	-0.822479	-0.738143	-0.124810
C	-0.340610	1.347138	-0.435374
O	0.449520	1.901426	-1.207241
C	-1.620332	1.974773	-0.089822
H	-2.349321	1.368917	0.477971
C	-1.968682	3.343537	-0.521481
O	-2.806375	3.439732	-1.628130
C	-2.240673	4.397851	0.505398
H	-2.515023	2.756558	-2.253977
H	-1.425149	4.437266	1.245365
H	-2.322473	5.383970	0.019701
H	-3.189333	4.211896	1.052439

T_1 minimum

C	0.192987	0.081081	0.104832
H	-0.540562	-0.604452	0.561467
H	0.843803	0.457333	0.911970
H	0.795894	-0.463763	-0.634566
C	-0.518394	1.234951	-0.571483
O	-0.399959	1.462227	-1.786614
C	-1.412658	2.073156	0.225550
H	-1.819669	1.667270	1.164825
C	-1.970522	3.328650	-0.363287
O	-2.757037	3.133568	-1.474503
C	-2.449720	4.420024	0.536617
H	-2.243693	2.513404	-2.026035
H	-1.681361	4.669784	1.285734
H	-2.681771	5.321114	-0.052479
H	-3.372140	4.126974	1.081115

T_2/T_1 MECI

C	0.025835	-0.045453	0.094403
H	-0.014714	0.024347	1.191248
H	1.081978	-0.088937	-0.222161
H	-0.462327	-0.982938	-0.222283
C	-0.670421	1.157083	-0.499575
O	-0.687142	1.183571	-1.819285
C	-1.268141	2.195573	0.248327
H	-1.211597	2.099740	1.335683
C	-1.921383	3.328912	-0.278790
O	-2.055960	3.562798	-1.621195
C	-2.541058	4.401230	0.556471
H	-1.629546	2.831556	-2.103419
H	-2.417225	4.187631	1.628330
H	-2.082610	5.382646	0.337359
H	-3.620476	4.493564	0.338015

9.2 CASSCF optimized geometries

S_0 minimum

C	0.005292	-0.000123	0.097958
H	-0.623412	-0.455826	0.865451
H	0.891765	0.402169	0.592233
H	0.304252	-0.757687	-0.623117
C	-0.748016	1.106732	-0.603753
O	-0.935517	1.054708	-1.806977
C	-1.232198	2.223544	0.218372
H	-1.027186	2.198973	1.278167
C	-1.921224	3.282231	-0.278056
O	-2.240212	3.436422	-1.562863
C	-2.414998	4.421517	0.561474
H	-1.896324	2.671598	-2.057633
H	-2.151480	4.289736	1.608958
H	-1.985949	5.357242	0.199981
H	-3.499584	4.500087	0.472934

S_2/S_1 MECI

C	-0.000782	-0.026728	0.028477
H	0.001900	-0.036773	1.118303
H	1.034356	-0.063448	-0.320746
H	-0.495773	-0.934991	-0.324947
C	-0.691310	1.188531	-0.502235
O	-0.761066	1.320696	-1.807710
C	-1.280397	2.214088	0.320143
H	-1.231024	2.118257	1.394584
C	-1.933413	3.363879	-0.251131
O	-1.984641	3.464230	-1.560347
C	-2.562894	4.460834	0.546159
H	-1.383463	2.412912	-1.913160
H	-2.465320	4.279079	1.616329
H	-2.095523	5.421315	0.314014
H	-3.625438	4.549443	0.305397

S_1 minimum

C	0.022350	-0.038681	0.126024
H	-0.015452	0.026591	1.211884
H	1.069699	-0.087015	-0.182688
H	-0.459454	-0.969829	-0.182855
C	-0.662774	1.148154	-0.479151
O	-0.649262	1.125034	-1.842560
C	-1.267020	2.194540	0.203676
H	-1.223488	2.118984	1.280758
C	-1.919143	3.323954	-0.297805
O	-2.082967	3.607773	-1.615725
C	-2.528513	4.379547	0.567909
H	-1.690681	2.927708	-2.176255
H	-2.396509	4.150819	1.624526
H	-2.073424	5.351944	0.362721
H	-3.598152	4.471800	0.362670

S_1 TS

C	1.533657	-1.984500	0.204754
H	0.872432	-2.682391	0.713944
H	2.394267	-1.780154	0.843693
H	1.897690	-2.449935	-0.712720
C	0.792218	-0.710686	-0.107124
O	1.586642	0.161110	-0.747401
C	-0.489757	-0.449099	0.239399
H	-0.995285	-1.242517	0.777288
C	-1.247419	0.797744	-0.034490
O	-1.947596	0.846437	-1.214135
C	-0.664313	2.128819	0.313535
H	-2.194521	-0.051502	-1.469686
H	-0.328074	2.144127	1.350734
H	0.197228	2.358529	-0.326506
H	-1.407171	2.914018	0.168715

S_1/S_0 MECI

C	-0.009941	0.018405	0.081311
H	-0.653627	-0.512940	0.781148
H	0.967213	0.162952	0.544210
H	0.130375	-0.600378	-0.806182
C	-0.603839	1.346914	-0.300658
O	0.064987	2.084476	-1.132991
C	-1.789114	1.876383	0.138175
H	-2.440477	1.410328	0.869882
C	-2.050964	3.206642	-0.458994
O	-2.914162	3.288716	-1.495775
C	-2.024553	4.457550	0.357804
H	-2.927830	2.434424	-1.952137
H	-1.136877	4.482405	0.988495
H	-2.026924	5.331622	-0.293681
H	-2.908457	4.515626	1.005092

T_1 minimum

C	0.197864	0.103426	0.157520
H	-0.555454	-0.687033	0.178488
H	0.487472	0.310874	1.189686
H	1.067355	-0.246677	-0.394814
C	-0.352509	1.348771	-0.503554
O	0.159094	1.810882	-1.512851
C	-1.521652	1.984423	0.084111
H	-2.019496	1.482460	0.910613
C	-2.100811	3.241572	-0.452147
O	-3.170400	3.099400	-1.300109
C	-2.275747	4.409813	0.467089
H	-2.955125	2.430238	-1.962596
H	-1.356226	4.608584	1.018922
H	-2.540280	5.297348	-0.108544
H	-3.078888	4.227242	1.191332

References

[1] Coussan, S., Ferro, Y., Trivella, A., Rajzmann, M., Roubin, P., Wieczorek, R., Manca, C., Piecuch, P., Kowalski, K., Włoch, M., Kucharski, S.A., Musiał, M.: Experimental and theoretical UV characterizations of acetylacetone and its isomers. The Journal of Physical Chemistry A **110**(11), 3920–3926 (2006)

[2] Nangia, S., Jasper, A.W., Miller, T.F., Truhlar, D.G.: Army ants algorithm for rare event sampling of delocalized nonadiabatic transitions by trajectory surface hopping and the estimation of sampling errors by the bootstrap method. The Journal of Chemical Physics **120**(8), 3586–3597 (2004) https://doi.org/10.1063/1.1641019

[3] Pons, O.: Bootstrap of means under stratified sampling. Electronic Journal of Statistics **1**, 381–391 (2007) https://doi.org/10.1214/07-EJS033

[4] Zhu, X., Thompson, K.C., Martínez, T.J.: Geodesic interpolation for reaction pathways. The Journal of Chemical Physics **150**(16), 164103 (2019)

[5] Squibb, R., Sapunar, M., Ponzi, A., Richter, R., Kivimäki, A., Plekan, O., Finetti, P., Sisourat, N., Zhaunerchyk, V., Marchenko, T., *et al.*: Acetylacetone photodynamics at a seeded free-electron laser. Nature communications **9**(1), 63 (2018)
